# Epidemiological Characteristics and Spatial-Temporal Distribution of Hand, Foot, and Mouth Disease in Chongqing, China, 2009–2016

**DOI:** 10.3390/ijerph15020270

**Published:** 2018-02-05

**Authors:** Li Qi, Wenge Tang, Han Zhao, Hua Ling, Kun Su, Hua Zhao, Qin Li, Tao Shen

**Affiliations:** 1 Chongqing Municipal Center for Disease Control and Prevention, Chongqing, No. 8, Changjiang 2nd Road, Yuzhong District, Chongqing 400042, China; cdcqili@126.com (L.Q.); wengetang@163.com (W.T.); molly_sunny2012@163.com (H.Z.); cdclinghua@126.com (H.L.); cqcdc_sk@sina.com (K.S.); cqcdczx@126.com (H.Z.); 2 Chinese Field Epidemiology Training Program, Chinese Center for Disease Control and Prevention, No. 27, Nanwei Road, Xicheng District, Beijing 100050, China

**Keywords:** hand, foot, and mouth disease, epidemiology, surveillance, seasonality

## Abstract

(1) *Objective*: Even with licensed vaccine for enterovirus 71 (EV71) put into market in 2016 in China, hand, foot, and mouth disease (HFMD) is still a threat for children’s health in Chongqing. We described the epidemiological characteristics and spatial–temporal patterns of HFMD in Chongqing from 2009 to 2016, in order to provide information and evidence for guiding public health response and intervention. (2) *Methods*: We retrieved the HFMD surveillance data from January 2009 to December 2016 from “National Disease Reporting Information System”, and then analyzed demographic and geographical information integrally. Descriptive analysis was conducted to evaluate the epidemic features of HFMD in Chongqing. The spatial–temporal methods were performed to explore the clusters at district/county level. (3) *Results*: A total of 276,207 HFMD cases were reported during the study period (total population incidence: 114.8 per 100,000 per year), including 641 severe cases (129 deaths). The annual incidence of HFMD sharply increased in even-numbered years, but remained stable or decreased in odd-numbered years. A semiannual seasonality was observed during April to July, and October to December in each year. The male-to-female ratios of the mild and severe cases were 1.4:1 and 1.5:1, with the median age of 2.3 years and 1.9 years, respectively. More than 90% of the cases were children equal to and less than 5 years old. High-incidence clustered regions included the main urban districts and northeast regions according to incidence rates comparison or space–time cluster analysis. A total of 19,482 specimen were collected from the reported cases and 13,277 (68.2%) were positive for enterovirus. EV71 was the major causative agent for severe cases, while other enteroviruses were the predominant serotype for mild cases. (4) *Conclusions:* The characteristics of HFMD in Chongqing exhibited a phenomenon of increasing incidence in two-year cycles and semiannual seasonality in time distribution. Children ≤5 years old, especially boys, were more affected by HFMD. EV71 was the major causative agent for severe cases. We suggest initiating mass EV71 vaccination campaigns among children aged 6 months to 5 years in Chongqing, especially in the main urban districts and northern regions, in order to reduce case fatality, and take integrated measurements for controlling and preventing HFMD attributed to other enteroviruses.

## 1. Introduction

Hand, foot, and mouth (HFMD) disease is an infectious disease caused by a group of enteroviruses [1], mainly coxsackievirus A16 (CVA16) and enterovirus 71 (EV71), and recently, coxsackievirus A6 (CVA6) is playing a more important role [2]. HFMD is typically characterized by fever, skin eruptions on hands, feet, buttocks, and vesicles/ulcer in the mouth. Most HFMD cases are mild and self-limited; however, some cases rapidly develop serious complications, such as meningitis and encephalitis, which can be fatal [3]. Enteroviruses are spread by various routes of transmission, including direct contact fluid from blisters, through the gastrointestinal tract, and the respiratory tract.

As one of the most serious epidemic areas, China was heavily burdened with HFMD. It was estimated that the incidence of HFMD was 1.2 per 1000 person years in the 2010–2012 period in China, and the disease was responsible for 500–900 reported deaths every year, mainly in children [4]. The 2016 Chinese yearbook of health statistics showed that incidence of HFMD ranked the first in the list of 39 notifiable infectious diseases, followed by tuberculosis and hepatitis. Chongqing is the largest municipality under direct control of the national government in China, located in the southwestern part of China. Previous studies about HFMD were mainly focused on characteristics of hospitalized cases [5,6], and lack of geographic information; therefore, the epidemiological characteristics and spatial–temporal patterns of HFMD in Chongqing were still unclear. EV71 vaccines have been licensed by the Chinese Food and Drug Administration in 2015, but not widely used in Chongqing yet.

We conducted this study to present the most comprehensive and updated epidemiological evidence of HFMD, and to detect spatial–temporal clusters in Chongqing, China, from 2009 to 2016. 

## 2. Materials and Methods

### 2.1. Data Collection

On 2 May 2008, HFMD was set statutorily notifiable as a Class C (third level of severity and importance of public health) infectious disease in mainland China. Since then, all HFMD cases diagnosed in hospitals were reported to “National Disease Reporting Information System” (NDRIS) within 24 h after diagnoses, including hospitalized and ambulatory cases, not only referred cases, but also self-presenting cases, according to the requirement of the law of the People’s Republic of China on Prevention and Treatment of Infectious Diseases. Previous studies showed that data collected during the first year was less reliable than those from more recent years, mainly because of improvements in surveillance and reporting process [4]; therefore, we excluded the data recorded in 2008 and analyzed the epidemiological data of HFMD cases in Chongqing from 1 January 2009 to 31 December 2016, from the NDRIS, which was stable in this period. The numbers of mid-year population were retrieved from bureau of census of Chongqing.

### 2.2. Case Definitions

The diagnosis of HFMD was carried out according to the criteria issued by National Health and Family Commission of the People’s Republic of China [7].

A clinical case of HFMD was defined as a patient with papular or vesicular rash on hands, feet, mouth, or buttocks, with or without fever.

A severe case was defined as HFMD accompanied with any of the following complications: aseptic meningitis, encephalitis, acute flaccid paralysis, pulmonary edema, hemorrhage, or cardiopulmonary failure. Otherwise, cases were categorized as mild cases.

A confirmed case was defined as a clinically diagnosed case with laboratory evidence of enterovirus infection (including EV71, CVA16, or other enteroviruses) detected by reverse transcription-polymerase chain reaction (RT-PCR), real-time RT-PCR, or virus isolation [7].

### 2.3. Specimen Collection and Laboratory Testing

According to the national guidelines [7], appropriate clinical specimens, including throat swab, rectal swab, fecal sample, vesicular fluid, and/or cerebrospinal fluid, were collected from the first batch of five clinical HFDM cases every month in each of the 39 districts or counties in Chongqing. We also attempted to collect specimens from all severe cases. Specimens were placed in 3 mL of sterile viral transport medium and sent to biosafety level two facilities for RT-PCR test within 48 h of collection, using commercially available pan-enterovirus, EV71, and CVA16 diagnostic kits, according to standardized protocols disseminated by the National Center for Disease Control and Prevention (CDC) [8]. Thus, the test results were classified into four categories: enterovirus negative, EV71 positive, CVA16 positive, or another enterovirus with unknown genotype.

### 2.4. Ethics Statement

This study was approved by the Ethics Committee of Chongqing Center for Disease Control and Prevention. All individual identifying information (including name, address, and telephone number, etc.) was anonymized and de-identified prior to analysis. Written informed consents were obtained from the cases or their parents/guardian before the clinical specimens were collected. 

### 2.5. Statistical Analysis

Descriptive analyses were performed and presented in percentage, median, and interquartile range (IQR) to present the demographic characteristics of HFMD cases. Significance of difference was initially assessed with Pearson’s chi-squared test or Kruskal–Wallis test, wherever appropriate. The *p* value < 0.05 was considered as statistically significant. SaTScan^TM^ software, version 9.1 using the Kulldorff method of retrospective space–time scan statistic based on a discrete Poisson model was used to detect HFMD clusters in individual districts/counties during the study period. The maximum spatial size of the cluster is 20% of the total population, and the maximum temporal size of the clusters is 6 months. The number of Monte Carlo simulations was set at 999.

## 3. Results

### 3.1. Epidemiological Characteristics of HFMD Cases

A total of 276,207 HFMD cases were reported during 2009–2016 in Chongqing, China, with an average annual incidence of 114.8 per 100,000 (ranged from 32.3 to 241.3). Of them, 641 (0.2%) were severe cases (Table 1). The median age of the mild cases was 2.3 years, with an interquartile range from 1.4 to 3.5 years, where 68.8% of the cases were under three years old and 91.3% were under five years old, respectively. The median age of severe cases was 1.9 years with an interquartile range from 0.9 to 2.9 years. Compared with mild cases, more severe cases occurred in the under five years old groups (95.2%) (χ^2^ = 140.9, *p* < 0.01). There was no significant difference between the proportion of cases under three between mild (68.8%) and severe cases (72.2%) (χ^2^ = 2.8, *p* = 0.093). The male-to-female ratios were 1.4:1 for mild cases, and 1.5:1 for severe cases, respectively. Regarding to the population classification, scattered children (children who do not reach the age of 3 years old to go kindergarten, or are taken care of by their family members) was the leading population, accounting for 63.8% and 87.8% of mild cases and severe cases respectively, followed by children in kindergarten (31.2% and 26.5%). The characteristics of reported HFDM cases were shown in Table 1.

Of the 641 severe cases, 512 cases recovered after treatment, but 129 cases died of pulmonary edema, hemorrhage, or cardiopulmonary failure.

### 3.2. Spatial–Temporal Clusters

**Figure 1:**
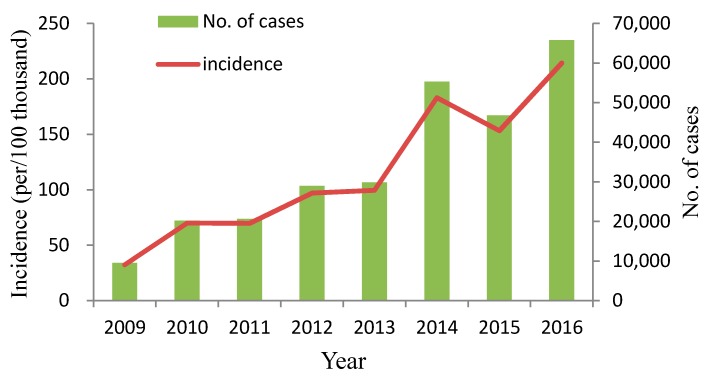
Annual incidence and number of cases of HFMD in Chongqing, China, 2009–2016.

**Figure 2:**
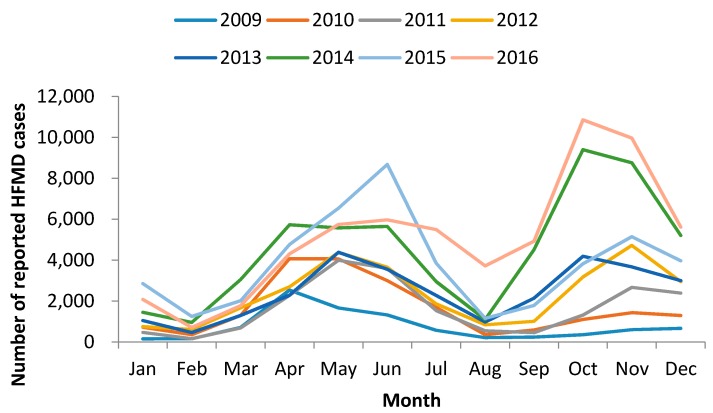
Monthly distribution of HFMD cases in Chongqing, China, 2009–2016.

The variations of yearly and monthly distributions are shown in Figure 1 and Figure 2. It is noteworthy that the annual incidence of HFMD sharply increased in even-numbered years (i.e., 2010, 2012, 2014, and 2016) and remained or decreased in odd-numbered years, which demonstrated a phenomenon of increasing incidence in two-year cycle in Chongqing. Besides, semiannual peaks of HFDM were observed during the study period. The peaks occurred from April to July, and October to December in each year, with the first peak higher than the second in most years, except for 2014 and 2016.

The variation of geographical distribution of HFMD indicated that main urban districts and northeast regions had higher incidence than other regions.

**Figure 3:**
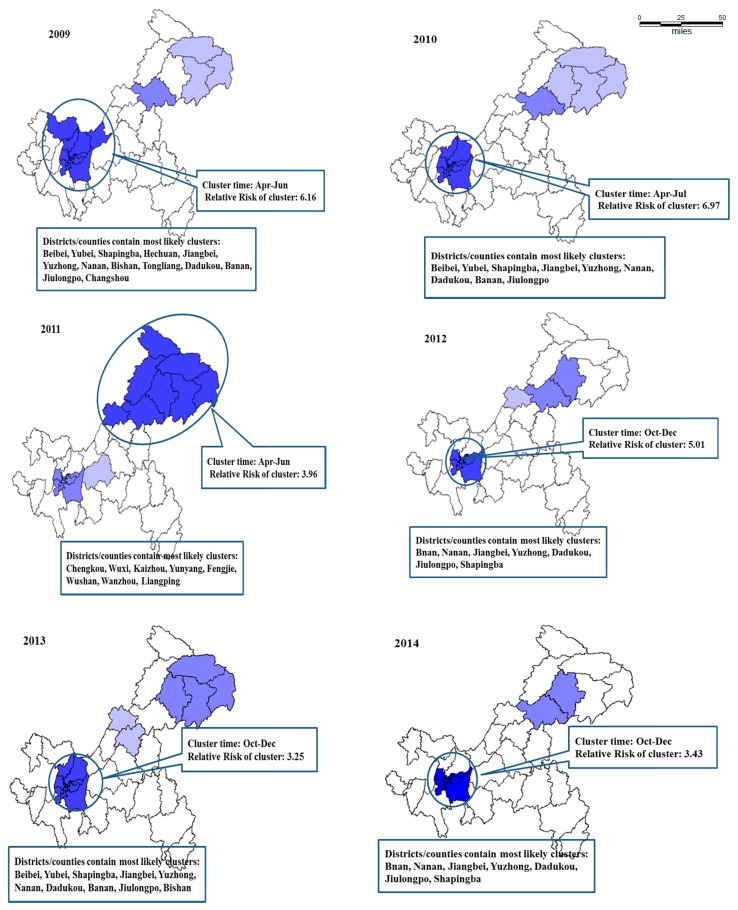

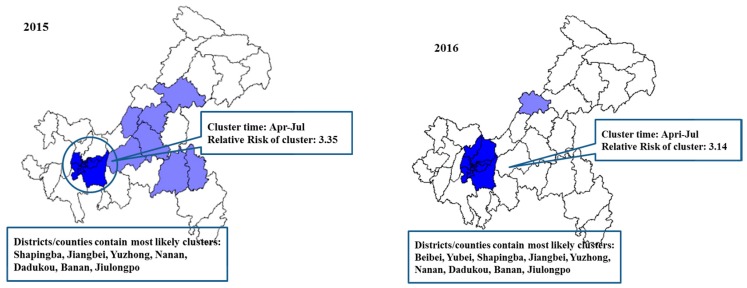
Spatial clustering of HFMD identified in Chongqing, China, from 2009 to 2016. Dark blue, the most likely clusters; light blue, the second most likely clusters.

**Figure 4:**
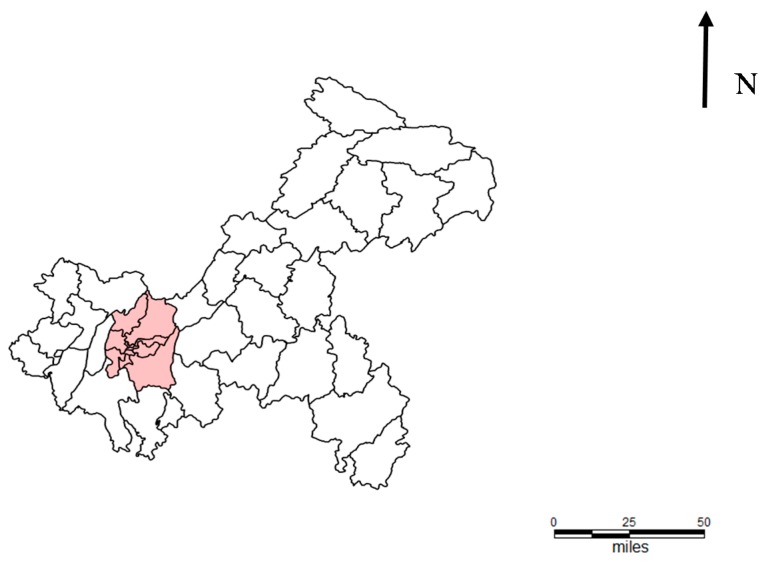
The nine main urban districts in Chongqing (Yuzhong district, Nanan district, Shapingba district, Jiangbei district, Yubei district, Jiu Longpo district, Beibei district, Banan district, and Dadukou district).

Table 2 listed the scanning results of most likely clusters for HFMD cases derived from the retrospective space–time cluster analysis. These showed that the most likely clusters (dark blue) were mainly located in nine main urban districts, except 2011: the most likely cluster areas in 2011 were the northeast regions (Figure 3 and Figure 4). The second most likely clusters (light blue) varied each year and spread all over the municipality (Figure 3). The cluster times were mainly April to June and September to December (Table 2).

### 3.3. Distribution of Pathogens’ Serotypes

Of 276,207 cases, a total of 19,482 (6.7%) specimens were collected, and 13,277 (68.2%) were detected as positive for enterovirus (Table 3).

**Figure 5:**
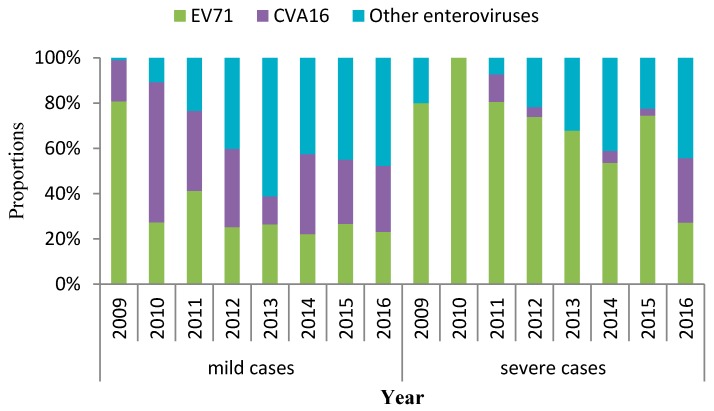
Proportions of enterovirus serotypes in laboratory-confirmed mild HFMD cases in Chongqing, China, 2009–2016. EV71: enterovirus A71; CVA16: coxsackie A16.

For mild cases, the predominant serotype was other enteroviruses since 2012 (accounting for 61.3% in 2012, 42.6% in 2013, 45.0% in 2014, 47.7% in 2015, and 40.7% in 2016), but specific genotype was not identified (Figure 5). In 2009 and 2011, a large percentage of EV71 was detected, and accounted for 80.7% and 41.2%, respectively. In 2010, the majority of the virus was CVA16 (61.9%).

The distribution of pathogen’ serotypes of the severe cases was significantly different from that of the mild cases. EV71 was the major causative agent until 2015, accounting for 72.2% of the severe HFMD cases. But other enteroviruses (44.3%) were dominant, taking place of EV71 (27.1%) in 2016 (Figure 5).

## 4. Discussion

Chongqing, the largest municipality under direct control of the national government in China, has experienced a continuous increase in incidence of HFMD from 2009 to 2016. The incidence was much higher than the national incidence, as well as that in many countries or regions over the same time period [4,8].

The epi-curve of HFMD in Chongqing exhibited a phenomenon of increasing incidence in a two-year cycle, similar to other provinces in China [9], which was not the same as the epidemic pattern in Taiwan [10], Singapore [11], Malaysia [12], and Japan [13], where it was shown that the epidemic pattern of HFMD occurred every 3 to 4 years. Besides, HFMD showed semiannual peaks of activity in Chongqing, which was also commonly observed in other southern provinces in China [4,14] and other countries, such as Vietnam [15], and Singapore [11], but different from annual epidemics in Japan [4,13]. Several factors have been proposed to explain the different seasonal patterns of HFMD in different regions, including the temperature, humidity, other meteorological factors, host susceptibility, population density, birth rate, and the environmental conditions [16,17,18,19,20,21,22,23,24,25,26]. In order to better understand the influential factors of HFMD and predict future occurrence of HFMD in Chongqing, future studies including these indicators should be considered in this area.

Even though strategies and measurements, such as symptoms surveillance in the gate of kindergartens and primary schools, timely isolation of cases, and daily disinfecting of toys and environment, were brought up for intervening the transmission of HFMD in Chongqing and all over China, the number of cases still increased in this seven-year period, which might be attributed to several influential factors, including improvement of the awareness of HFMD among the physicians in hospital and the parents of children, which might lead to the increase in the number of hospital visits, diagnoses, and reporting; these measures were not strictly complied with, since some facilities such as private kindergartens want to make profits by keeping more children in, and did not isolate the HFMD-infected child by sending him/her home, which will lead to sustained transmission of HFMD in kindergartens, and they might not disinfect the toys or environment regularly according to guidelines for reducing cost.

The observed age profile of infection in this study showed sharp discordance among the different age groups. More than 90% of cases were concentrated in children less than five years old, and especially those under three years old; the median age of severe cases was younger than mild cases, in line with some other reports [4,26,27,28]. One of the important explanations of discordance among the different age groups was that the levels of antibodies against enteroviruses were increased by age, because of asymptomatic infection [29,30]. Consistent with previous studies [4,28,31], males were more frequently infected than females, and so was the ratio of severe cases; this phenomenon may be related to some factors including male susceptibility at the host genetic level, host immune status, behavior pattern, and it may also be due to reporting bias [4].

The districts/counties with high-incidence showing clustering were consisted of the main urban districts and northeast counties, according to incidence rates comparison or spatial–temporal cluster analysis. Several possible explanations were the density of population, the suitable meteorological conditions, the socioeconomic status, availability of health care, and the diagnosis level in these regions, which needed to be further researched.

Regarding the etiology, in line with previous reports [4], EV71 was the major causative agent of severe cases of HFMD. One of the most important explanations might be that EV71 is more virulent than other enteroviruses. Thus, the serological distribution of the enteroviruses can be a predictor for the early warning of epidemics of fatal cases, and we also consider that the principal and most promising strategy of controlling and preventing severe and fatal HFMD cases is to prevent EV71 circulation in children through mass EV71 vaccination [32].

Given the public health impact and epidemiological characteristic of HFMD in Chongqing, and EV71, with which vaccines for prevention can decrease the fraction of total HFMD cases [32], integrated strategies and measurements are recommended, including improving awareness of the importance of hand-washing for preventing HFMD, regularly cleaning and disinfecting the toys, appliances, and environment in kindergartens [7], timely isolation of the patient, and allowing the child to come back kindergarten only if recovered.

To the best of our knowledge, this is the most comprehensive study of HFMD up to now in Chongqing, China. The findings can be helpful for the control and prevention of HFMD epidemics in this area. Moreover, our results will serve as a pre-vaccination baseline against which future interventions can be evaluated.

Some limitations of this study deserve mention. First, only 6.7% of the reported HFMD cases were tested for the pathogens associated with the infection, due to that the purpose of laboratory testing is to determine the predominant virus circulation in Chongqing, rather than to verify each case with HFMD. Second, we did not determine the serotype enteroviruses beyond CVA16 and EV71, especially CVA6, which was observed to have increased dramatically in recent years [33,34,35,36]; further studies are needed to identify other enteroviruses in future, and then accelerate the development of vaccines against those pathogens, which will play an important role in the prevention of HFMD. Third, the HFMD surveillance system has only been operated for around eight years; a longer time for trend analysis is probably needed in future, and this surveillance system is a passive information collecting system, and might be underreporting HFMD cases, especially mild cases, which could lead to underestimation of the incidence rate.

## 5. Conclusions

The epidemics of HFMD in Chongqing exhibited a phenomenon of increasing incidence in two-year cycles, and semiannual seasonality. Children ≤ 5 years old were more susceptible to HFMD. EV71 was the major causative agent for severe cases. We suggest initiating a mass EV71 vaccination program for infants and young children in Chongqing, especially in the main urban districts and northern regions, in order to reduce the overall HFMD burden, and take integrated measurements for controlling and preventing HFMD attributed to other enteroviruses.

## Figures and Tables

**Table 1:** The demographic characteristics of hand, foot, and mouth disease (HFMD) cases in Chongqing, 2009–2016.

	Total	2009	2010	2011	2012	2013	2014	2015	2016
	(*n* = 276,207)	(*n* = 9558)	(*n* = 20,209)	(*n* = 20,641)	(*n* = 28,995)	(*n* = 29,883)	(*n* = 55,320)	(*n* = 46,757)	(*n* = 64,844)
	*n* (%)	*n* (%)	*n* (%)	*n* (%)	*n* (%)	*n* (%)	*n* (%)	*n* (%)	*n* (%)
**Age (year)**									
less than 1 year	25,929 (9.4)	660 (6.91)	1397 (6.9)	1551 (7.5)	2703 (9.3)	3454 (11.6)	4671 (8.4)	4224 (9.0)	7269 (11.2)
1~	86,149 (31.2)	1099 (11.5)	5175 (25.6)	5297 (25.7)	8467 (29.2)	10,413 (34.9)	17,609 (31.8)	14,628 (31.3)	23,461 (36.2)
2~	64,142 (23.2)	2662 (27.9)	5380 (26.6)	5173 (25.1)	7091 (24.5)	6557 (21.9)	13,410 (24.2)	10,335 (22.1)	13,534 (20.9)
3~	53,560 (19.4)	2437 (25.5)	4445 (22.0)	4483 (21.7)	5910 (20.4)	5120 (17.1)	10,435 (18.9)	9560 (20.5)	11,170 (17.2)
4~	24,049 (8.7)	1399 (14.6)	1870 (9.3)	2110 (10.2)	2608 (9.0)	2226 (7.5)	4784 (8.7)	4084 (8.7)	4968 (7.7)
5~	10,022 (3.6)	655 (6.9)	690 (3.4)	935 (4.5)	1041 (3.6)	962 (3.2)	2013 (3.6)	1845 (3.9)	1881 (2.9)
more than 6 years	12,356 (4.5)	646 (6.8)	1252 (6.2)	1092 (5.3)	1175 (4.1)	1151 (3.9)	2398 (4.3)	2081 (4.5)	2561 (3.9)
**Gender**									
Male	162,934 (59.0)	5739 (60.0)	12,096 (59.9)	12,612 (61.1)	17,647 (60.9)	17,792 (59.5)	32,216 (58.2)	27,309 (58.4)	37,523 (57.9)
Female	113,273 (41.0)	3819 (40.0)	8113 (40.1)	8029 (38.9)	11,348 (39.1)	12,091 (40.5)	23,104 (41.8)	19,448 (41.6)	27,321 (42.1)
Sex ratio	1.44	1.50	1.49	1.57	1.56	1.47	1.39	1.40	1.37
**Population classification**									
In kindergarten	86,339 (31.2)	3868 (40.5)	8123 (40.2)	7186 (34.8)	9604 (33.1)	8087 (27.1)	17,053 (30.8)	14,965 (32.0)	17,453 (20.6)
Scatter children *	176,858 (63.8)	4920 (51.5)	11,050 (54.7)	12,088 (58.6)	17,760 (61.3)	20,329 (68.0)	35,611 (64.4)	29,545 (63.2)	45,555 (52.7)
In primary school	7504 (2.7)	347 (3.6)	719 (3.6)	727 (3.5)	811 (2.8)	755 (2.5)	1459 (2.6)	1161 (2.5)	1525 (1.8)
Others	5506 (2.3)	423 (4.4)	317 (1.6)	640 (3.1)	820 (2.8)	712 (2.4)	1197 (2.2)	1086 (2.3)	311 (0.4)
Severe cases	641	14	42	73	30	88	92	192	110

Note: * Scattered children are defined as children who do not reach the age of 3 years old to go kindergarten or taken care of by their family members.

**Table 2:** The scanning results of space–time cluster analysis for HFMD cases from Chongqing, 2009–2016.

Year	Districts/Countries (*n*)	Cluster Centers/Radius	Time (Month)	Observed Cases (*n*)	Expected Cases (*n*)	RR	LLR	*p*-Value
2009	13	(29.87N, 106.49E)/64.12 km	April–June	4077	1039	6.16	3165.487	0.001
2010	9	(29.45N, 106.72E)/52.31 km	Apr–July	8667	1987	6.97	7526.839	0.001
2011	8	(31.92N, 108.77E)/170.97 km	Apr–June	3571	1037	3.96	2055.819	0.001
2012	7	(29.45N, 106.72E)/38.52 km	October–December	5524	1304	5.01	4098.742	0.001
2013	10	(29.61N, 106.37E)/39.48 km	October–December	5931	2092	3.25	2599.343	0.001
2014	7	(29.45N, 106.72E)/38.52 km	September–December	9960	3329	3.43	4729.999	0.001
2015	7	(29.45N, 106.72E)/38.52 km	April–July	7765	2649	3.35	3567.425	0.001
2016	9	(29.45N, 106.72E)/52.31 km	June–October	23,788	10,227	3.14	8444.554	0.001

Note: LLR Log Likelihood Ratio; RR Relative risk.

**Table 3:** The distribution of pathogens’ serotypes of mild and severe HFMD cases in Chongqing, 2009–2016.

Year	Mild Cases				Severe Cases				χ^2^	*p*-Value *
	Specimen	EV71	CVA16	Other Enterovirus	Specimen	EV71	CVA16	Other Enterovirus		
2009	298	155	35	2	14	4	0	1	6.1 **	0.078
2010	1418	246	558	97	42	22	0	0	49.9 **	<0.01
2011	2646	717	615	409	73	33	5	3	25.4	<0.01
2012	2284	397	545	633	30	17	1	5	28.8	<0.01
2013	2568	474	222	1100	88	40	0	19	50.6	<0.01
2014	4102	680	1084	1307	92	30	3	23	38.1	<0.01
2015	2734	525	561	887	191	96	4	29	136.6	<0.01
2016	2791	481	608	993	111	19	20	31	0.7	0.722

* *p*-value for comparison between the distribution of pathogen’ serotypes of the severe cases and that of the mild cases every year. ** Fisher’s exact test.
